# Refractory Gingival Enlargement: A Critical Oral Clue to Early‐Stage Granulomatosis With Polyangiitis—A Case Report and Literature Review

**DOI:** 10.1155/crid/8443058

**Published:** 2026-01-21

**Authors:** Sarwer Biplob, Rahmi Amtha, Najla Nadiah, Rachendra Pratama

**Affiliations:** ^1^ Oral and Maxillofacial Surgery, Bangabandhu Sheikh Mujib Medical University, Dhaka, Dhaka Division, Bangladesh, bsmmu.edu.bd; ^2^ Oral Medicine Department, Universitas Trisakti Fakultas Kedokteran Gigi, West Jakarta, Jakarta, Indonesia; ^3^ Oral and Maxillofacial Department, Universitas Trisakti Fakultas Kedokteran Gigi, West Jakarta, Jakarta, Indonesia

**Keywords:** granulomatosis with polyangiitis (GPA), immunosuppression, strawberry gingivitis, Wegener′s granulomatosis

## Abstract

**Introduction:**

Granulomatosis with polyangiitis (GPA), formerly known as Wegener′s granulomatosis, is a rare, systemic small‐vessel vasculitis characterized by necrotizing granulomatous inflammation. It classically affects the upper airway, lungs, and kidneys. Oral manifestations are infrequent, occurring in approximately 6%–13% of cases, and represent the initial presentation in only about 2%.

**Case Presentation:**

A 55‐year‐old female was presented with a two‐month history of painful, bleeding gingival hyperplasia unresponsive to routine periodontal therapy. Intraoral exam revealed “strawberry gingivitis”—diffuse, friable, erythematous granular gingival enlargement. Comprehensive workup showed elevated inflammatory markers and positive cytoplasmic ANCA (c‐ANCA). Gingival biopsy demonstrated granulomatous inflammation with necrotizing vasculitis, confirming localized GPA confined to oral tissues. Multidisciplinary management was initiated with systemic immunosuppression (oral azathioprine and deflazacort) and topical therapy, alongside optimization of the patient’s comorbid conditions. An antifungal and antibiotic prophylaxis regimen was also provided.

**Outcomes:**

Following the commencement of therapy, the gingival lesions exhibited complete resolution within 3 weeks. Over a 12‐month follow‐up period, there was no evidence of progression to other organ involvement. The concurrent management of the patient′s diabetes and hypothyroidism was instrumental in facilitating optimal healing and overall patient recovery.

**Conclusions:**

This case distinctly underscores that GPA can present solely with oral symptoms, making early recognition by dental clinicians paramount for prompt diagnosis. The presence of “strawberry gingivitis” serves as a crucial, pathognomonic clinical sign. We propose a diagnostic and management protocol for oral GPA to improve patient outcomes.

## 1. Introduction

Granulomatosis with Polyangiitis (GPA) is an uncommon autoimmune vasculitis characterized by necrotizing granulomas and pauci‐immune vasculitis of small to medium‐sized vessels [1]. It predominantly affects the upper and lower respiratory tracts and kidneys, and was historically known as Wegener′s granulomatosis until the 2012 Chapel Hill Consensus, which renamed the disease to GPA. The annual incidence is low (approximately 3–14 per million) with no strong gender predilection [2]. GPA is part of the ANCA‐associated vasculitis spectrum, most often associated with cytoplasmic ANCA against proteinase‐3 (c‐ANCA/PR3) [3].

Head and neck involvement occurs in the majority of GPA patients, but oral manifestations are relatively rare. Oral lesions are reported in only about 6%–13% of GPA [1, 3]. Moreover, the oral cavity is the initial site of presentation in a small minority (on the order of 2%–6% of cases) [3, 4]. Among oral signs, “strawberry gingivitis” is considered a hallmark feature of GPA, it presents as a reddish‐purple, friable granular hyperplasia of the gingiva with scattered petechiae, resembling the surface of a strawberry [3]. When present, this finding is almost pathognomonic for GPA [5]. However, because of its rarity, clinicians may overlook strawberry gingivitis or confuse it with more common gingival conditions. Indeed, oral GPA lesions are often misdiagnosed as periodontal disease or other oral pathologies, leading to delays in correct diagnosis and treatment [6]. Early recognition is critical, untreated GPA can progress rapidly, with an estimated 85% one‐year mortality from renal or respiratory failure in the pre‐treatment era [4].

This report describes a rare case of GPA presenting solely with gingival manifestations. We outline the patient′s clinical course and successful interdisciplinary management. In addition, we provide a comprehensive literature review of oral manifestations of GPA, including prevalence, differential diagnoses, pathophysiology, and treatment strategies. Finally, we propose a systematic clinical approach for suspected oral GPA. The aim is to assist oral health providers in promptly identifying and managing this potentially lethal disease [6].

## 2. Case Presentation

A 55‐year‐old female patient was presented to the dental clinic with a chief complaint of gum swelling and bleeding for 2 months. She noted progressive enlargement of the gums in both upper and lower jaws, spontaneous bleeding, and pain on brushing. The patient had been treated initially with scaling and oral antibiotics by her general dentist, with no improvement. Her medical history was significant for Type 2 diabetes mellitus, hypothyroidism, nonalcoholic fatty liver disease, and chronic insomnia. She had no known history of autoimmune disease. There was no exposure to medications known to cause gingival overgrowth (such as phenytoin or cyclosporine). Family history was unremarkable. The patient′s diabetes and thyroid conditions were sub optimally controlled at presentation (HbA1c was 7.5%, TSH mildly elevated), which can contribute to periodontal inflammation. However, the rapid progression and severity of her gingival changes were atypical for simple uncontrolled diabetes‐related periodontal disease.

**Figure 1:**
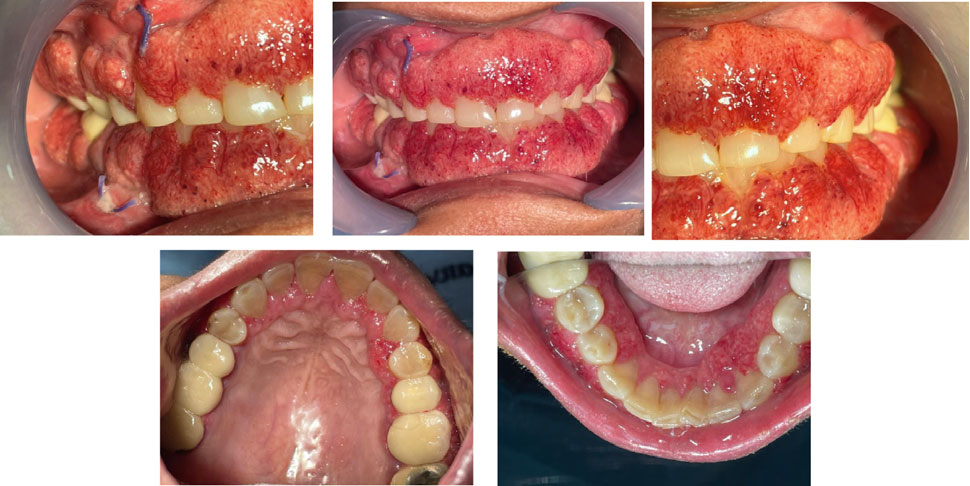
Preoperative intraoral presentation of gingival hyperplasia on Day 1.

On extraoral examination, there was no facial swelling, lymphadenopathy, or skin lesion. Intraoral examination revealed a diffuse gingival enlargement involving both the maxillary and mandibular arches (buccal and palatal/lingual aspects). The gingiva was markedly erythematous, friable, and had a granular or pebbled surface texture (Figure 1). The enlargement was most pronounced in the anterior regions. Light palpation induced bleeding. The appearance was highly consistent with so‐called “strawberry gingivitis,” a rare but pathognomonic manifestation of GPA [1]. The reminder of the oral mucosa showed no significant lesions aside from mild palatal petechiae. Several teeth (especially the maxillary incisors) exhibited Grade I mobility, likely due to inflammatory periodontal support loss. There was no purulent discharge, periodontal abscess, or deep periodontal pockets to suggest purely pyogenic periodontal disease. Given the dramatic gingival findings, an extensive differential diagnosis was considered (including leukemia, granulomatous disorders, and vasculitis). At this stage, an incisional biopsy of the gingiva was planned for definitive diagnosis. Informed consent was obtained from the patient prior to all procedures.

Baseline laboratory tests were ordered. The patient′s complete blood count showed mild anemia (hemoglobin 10.9 g/dL) and slight leukocytosis (11.2 × 10^9^/L) with neutrophil predominance, changes consistent with inflammation. Platelets were elevated (510 × 10^9^/L), and the erythrocyte sedimentation rate (ESR) was 72 mm/h (high). These findings suggested an active inflammatory or myeloproliferative process; importantly, no blast cells or atypical cells were seen on the blood smear, arguing against leukemia. Given the suspicion for vasculitis, serologic tests for ANCA were sent.

### 2.1. Investigations

An incisional biopsy was taken from the maxillary anterior gingiva under local anesthesia. Histopathological examination revealed hyperplastic stratified squamous epithelium with pseudoepitheliomatous hyperplasia (benign reactive acanthosis). The underlying connective tissue stroma was densely infiltrated with chronic inflammatory cells, including lymphocytes and plasma cells, along with clusters of neutrophils. Numerous small blood vessels showed necrotizing vasculitis—fibrinoid necrosis of vessel walls with neutrophil debris (leukocytoclastic vasculitis) was evident. Surrounding these vessels and dispersed in the stroma were foci of granulomatous inflammation with multinucleated giant cells. Areas of “geographic” necrosis (irregular zones of necrosis with palisaded histiocytes) were also noted. No evidence of dysplasia or malignancy was present. Special stains for infectious organisms were performed, including periodic acid—Schiff and Grocott′s methenamine silver stains showed no fungal forms, and acid‐fast bacilli stain was negative for mycobacteria. These histologic findings granulomas with necrotizing vasculitis, strongly pointed to GPA as the diagnosis in the appropriate clinical context.

The serologic workup returned with a positive c‐ANCA (high titer antiproteinase‐3 antibodies). This provided additional support for GPA. Antinuclear antibody was negative, and rheumatoid factor was normal, making alternative rheumatologic diseases unlikely. A chest radiograph and high‐resolution computed tomography of the chest were obtained to screen for pulmonary involvement; these showed clear lung fields with no nodules or infiltrates. Urinalysis and renal function tests were within normal limits (no hematuria, proteinuria, or rise in creatinine), suggesting no renal involvement at that time.

Collectively, the biopsy and serology confirmed the diagnosis of GPA localized to the gingiva. There was no clinical or radiographic evidence of active disease in the lungs, kidneys, or sinus nasal tract. Therefore, the case was classified as localized (limited) GPA presenting with oral manifestations.

### 2.2. Treatment

The therapeutic approach was two‐pronged including induce remission of the vasculitic process and address local oral care and risk factors. For systemic therapy, given the patient′s localized disease and multiple comorbidities, we selected a precisely defined treatment protocol. The regimen included Cps. cyclosporine 50 mg twice daily for the initial 7 days, along with T. deflazacort 30 mg/day initially (a corticosteroid) and T. azathioprine (started at 50 mg/day and titrated to 100 mg/day as tolerated). Following the initial 7‐day period, Cps. cyclosporine was discontinued, and therapy continued with T. deflazacort and T. azathioprine.

In addition to immunosuppression, prophylactic antimicrobial measures were implemented. Cps. doxycycline 100 mg daily was commenced. The rationale for this antimicrobial treatment lies in its efficacy against *Staphylococcus aureus*, a critical consideration given the established association between chronic nasal staphylococcal colonization and GPA relapse, while also offering auxiliary treatment for concurrent periodontal pathogens. A systemic antifungal, T. voriconazole 200 mg twice daily, was prescribed empirically to prevent fungal overgrowth in the context of steroid use and oral lesions. The T. voriconazole was planned for a short course (4 weeks), whereas the oral lesions healed, and the patient was monitored for hepatic side effects given her NAFLD.

Locally, the patient was advised on improved oral hygiene measures to reduce secondary bacterial load on the inflamed gingiva. The local regimen included a 0.2% chlorhexidine gluconate mouth rinse twice daily and a povidone‐iodine oral swab once daily to antiseptically cleanse the gingiva. Gentle toothbrushing with an ultrasoft toothbrush was encouraged to keep plaque levels low without traumatizing the friable tissue. We also prescribed a topical steroid dental paste (0.05% clobetasol) to apply to the gingiva twice daily for the first 2 weeks, in order to provide direct anti‐inflammatory effect in the oral lesions. However, the use of topical steroid was cautious and brief due to the already ongoing systemic steroid and concern for oral candidiasis (for which voriconazole was administered prophylactically). Pain was managed with periodic acetaminophen; no NSAIDs were given to avoid any additive renal risk.

**Figure 2:**
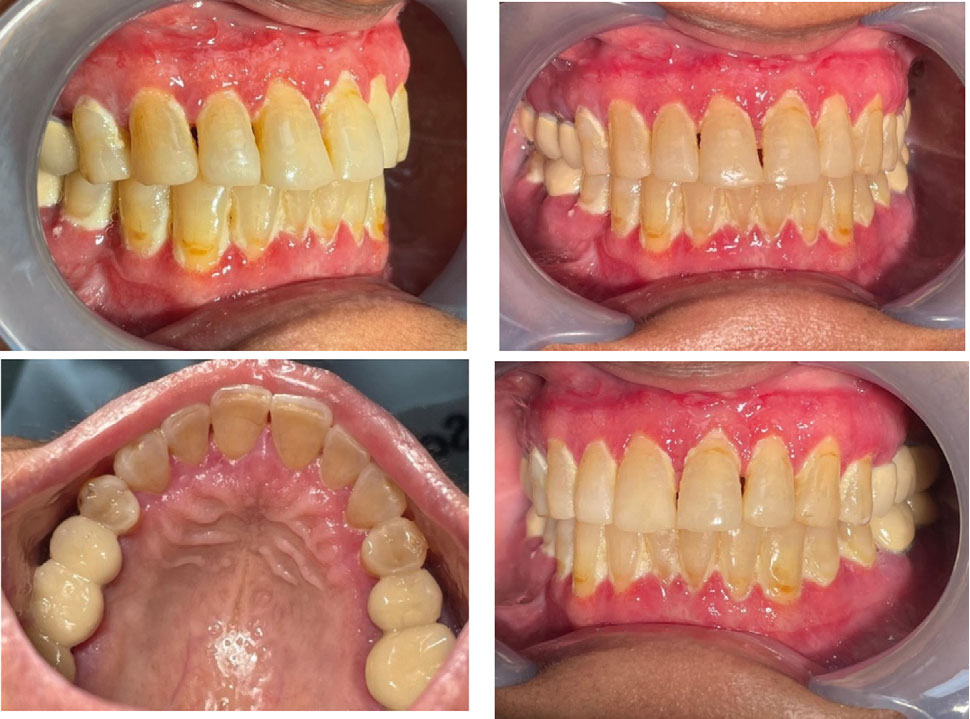
Intraoral healing after 14 days of immunosuppressive and topical therapy.

The patient′s response was closely monitored. Within 2 weeks of therapy initiation, she reported significant reduction in gingival swelling and bleeding. By the third week, the “strawberry” appearance of the gingiva had largely subsided, the tissue was pinker and firmer, with only mild residual hyperplasia, as shown in Figure 2. Repeat blood tests 1 month into therapy showed improvement: ESR dropped to 25 mm/h, and CRP normalized. Her c‐ANCA titer also decreased (from 1:640 to 1:160). These findings correlated with clinical remission of the oral disease.

The T. deflazacort dosage was progressively reduced over a 3‐month period, reaching a final maintenance dose of 6 mg/day. T. azathioprine was continued as maintenance therapy (100 mg/day) with plans for at least 18 months of therapy, barring any adverse effects or relapse. The patient′s diabetes was managed with adjusted insulin doses throughout, and notably her glycemic control improved as steroid doses were reduced. T. voriconazole was discontinued after 1 month as planned (no signs of fungal infection appeared). Cps. doxycycline was continued for 2 months and then stopped once the gingiva had completely healed and ANCA titers declined (to minimize long‐term antibiotic use).

**Figure 3:**
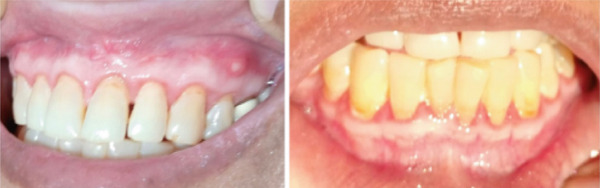
Follow‐up intraoral images after 10 months.

The patient continued to be closely monitored under the long‐term maintenance regimen. At the 10‐month follow‐up, total clinical resolution of the gingival hyperplasia was observed, indicating a significant improvement and sustained remission of the oral GPA, as shown in Figure 3. The patient reported immense satisfaction, noting that the resolution of the oral lesions completely restored her ability to eat and speak comfortably, significantly improving her quality of life.

## 3. Discussion and Literature Review

This case of GPA presenting solely as localized oral manifestations offers valuable insights and underscores several critical clinical lessons. While GPA predominantly affects the nasal tract and lungs, accounting for up to 90% of cases, this presentation highlights the importance of recognizing unusual initial signs [6]. Oral lesions are uncommon, occurring in only 6%–13% of GPA patients during the disease course, and are the initial manifestation in a mere 2%–6% of cases. This rarity, combined with their nonspecific nature, makes primary oral GPA a significant diagnostic challenge. Our patient′s gingival findings, specifically the classic “strawberry gingivitis,” were the sole initial manifestation. This underscores the need for clinicians, to maintain a high index of suspicion [3].

A delay in diagnosis is unfortunately common in such scenarios, with an average diagnostic delay of 4–5 months reported for limited disease. Our patient′s condition was initially misdiagnosed as a benign periodontal problem, a common pitfall. Fortunately, prompt recognition of the atypical gingival changes by the dental team, coupled with an early biopsy, led to a diagnosis within 2 months of symptom onset. This timely intervention likely prevented progression to systemic involvement, which is crucial given the aggressive nature of untreated GPA and its historical 85% 1‐year mortality rate from renal or respiratory failure. We strongly advocate for including GPA in the differential diagnosis of unexplained granulomatous gingival lesions.

“Strawberry gingivitis,” characterized by hyperplastic, erythematous, granular gingiva with pinpoint hemorrhages, is the most frequent oral manifestation of GPA, observed in approximately 60% of reported oral cases [3]. It often involves the interdental papillae and marginal gingiva, typically in the maxilla (though it may be generalized) [7]. While it often affects interdental papillae and marginal gingiva, particularly in the maxilla, it can be generalized. Patients commonly experience gingival bleeding, pain, and tooth mobility due to the underlying inflammation [7]. Other, less specific, oral manifestations can include mucosal ulcerations, localized necrotizing lesions, chronic sinus tracts, osteonecrosis of the jaw, and parotid swelling [8]. Crucially, oral lesions can precede systemic signs by weeks to months, necessitating a vigilant approach.

The patient was diagnosed with GPA, localized to the oral cavity (gingival involvement). This diagnosis was supported by the triad of clinical features (strawberry gingivitis), histopathological findings (necrotizing granulomatous vasculitis on gingival biopsy), and serology (positive c‐ANCA). Importantly, no other cause for the gingival lesions was found, and the presentation fulfilled the American College of Rheumatology criteria and Chapel Hill definitions for GPA [2]. The diagnosis was explained to the patient, emphasizing that although her disease was currently confined to the gums, GPA is systemic and could involve other organs in the future. A multidisciplinary treatment plan was formulated with involvement of rheumatology, oral medicine, and the patient′s primary care physician (for diabetes and thyroid management).

The differential diagnosis for diffuse gingival hyperplasia with a granulomatous or hemorrhagic appearance is extensive. Oral GPA lesions can mimic more common conditions, requiring careful exclusion, including hematologic malignancies (like leukemic gingivitis), other granulomatous diseases (such as sarcoidosis), various infectious causes, and drug‐induced gingival overgrowth (such as phenytoin, cyclosporine, and calcium channel blockers) [8]. A timely biopsy is paramount. In suspected cases, a comprehensive systemic work‐up (including complete blood count, inflammatory markers, ANCA testing, and imaging) should run in parallel with the exclusion of local causes. The combination of characteristic histopathology and a positive ANCA typically confirms the diagnosis. Multidisciplinary input from oral pathologists, dentists, and medical specialists is crucial for accurate diagnosis and management [7, 9].

GPA is an ANCA‐associated vasculitis, with c‐ANCA (anti‐proteinase‐3) being the most common autoantibody [3]. These autoantibodies are pathogenic, leading to aberrant neutrophil activation, endothelial damage, and granulomatous inflammation [10]. The histopathologic hallmark of GPA includes necrotizing vasculitis, granulomatous inflammation, and geographic necrosis. Oral biopsies often reveal an inflammatory infiltrate with neutrophils, lymphocytes, multinucleated giant cells, palisading histiocytes around necrotic areas, and fibrinoid necrosis within vessel walls [4, 8]. Pseudoepitheliomatous hyperplasia is also common [8].

Our patient′s strongly positive c‐ANCA supported the biopsy findings. Imaging of the chest and sinuses is recommended even in asymptomatic patients to detect subclinical involvement, as GPA can manifest in other organs. Our patient′s normal chest *X*‐ray and intact renal function were consistent with truly localized disease. Exclusion of other differential diagnoses through complete blood count (to rule out leukemia), negative fungal or bacterial cultures, and absence of antinuclear antibodies is also vital. The definitive diagnosis of GPA is usually established by combining characteristic histology with positive ANCA, though rare ANCA‐negative does not rule out the disease, particularly in early cases [8]. The study by Nico et al. demonstrated this by presenting a case of typical “strawberry gingivitis” with negative ANCA results that still responded to immunosuppressive therapy, reinforcing the diagnosis. Histopathologically, the diagnosis of oral GPA lesions is often challenging as necrotizing granulomas and vasculitis may be absent. However, the presence of epithelial changes such as pseudoepitheliomatous hyperplasia and intraepithelial neutrophilic microabscesses, can serve as important diagnostic features for the oral lesions of GPA [11].

The management of oral GPA aligns with the guidelines of systemic GPA treatment, tailored to the extent of the disease. Prompt initiation of immunosuppressive therapy is critical due to the potentially fatal nature of untreated GPA [4]. Remission induction typically involves corticosteroids combined with cyclophosphamide or rituximab, particularly for severe or generalized disease [12, 13]. For limited or nonsevere cases, methotrexate combined with steroids can be an alternative. Glucocorticoids are a cornerstone for rapid inflammation control cases [8]. High‐dose corticosteroid was necessary to control active inflammation; deflazacort was used instead of prednisone for its potentially favorable side effect profile regarding glucose metabolism (relevant to her diabetes). Furthermore, azathioprine, an antimetabolite immunosuppressant was selected over cyclophosphamide or methotrexate to reduce cytotoxic exposure, given that her GPA did not appear life‐threatening or widely disseminated at presentation. Rheumatology consultation concurred with this conservative immunosuppressive strategy, with a plan to escalate therapy if any systemic involvement emerged. The patient′s endocrinologist adjusted her insulin regimen to anticipate steroid‐induced hyperglycemia, and her levothyroxine dose was optimized for hypothyroidism, as uncontrolled endocrine issues could hinder recovery. Fortunately, she achieved remission with the above regimen, so more aggressive agents were not required. The patient tolerated azathioprine well; liver function tests and blood counts remained within safe ranges on serial monitoring.

After induction of remission, maintenance therapy is required to prevent relapse. Maintenance therapy, typically with azathioprine, methotrexate, or rituximab, is essential to prevent relapse and is usually continued for 12–24 months or longer [12]. Our patient′s localized disease allowed for initiation with azathioprine alongside a tapering corticosteroid course, acting as both an induction and maintenance agent. This approach reflects the increasing trend toward personalized therapy based on disease extent and patient factors.

Case reports on oral GPA treatment highlight a range of successful approaches, with most patients ultimately requiring systemic therapy. However, newer biologic therapies like rituximab are increasingly used, especially in relapsing or refractory cases [3]. Our patient′s successful remission with a less aggressive regimen (azathioprine and steroids) aligns with findings from studies by Subramanya et al. This particular case of localized oral GPA demonstrated successful remission without the use of cyclophosphamide or other highly cytotoxic agents, underscoring the potential for less intensive immunosuppression in select limited cases. Nevertheless, close monitoring remains paramount for potential systemic progression of the disease [8].

Adjunctive therapies, such as prophylaxis against pneumocystis pneumonia was discussed, but ultimately not started due to the patient′s sulfa allergy; instead, we monitored closely for signs of pneumocystis and counseled her on prompt reporting of respiratory symptoms. Our patient, with moderate immunosuppression from dual therapy, existing diabetes, and persistent oral lesions, received individualized antifungal (voriconazole) and antibacterial (doxycycline) prophylaxis. This was a precautionary step given the severe gingival inflammation and the need to rule out any superadded fungal infection (no fungus was seen on biopsy, but cultures were pending at the time and came back negative).

The advent of aggressive immunotherapy has dramatically improved GPA survival, with remission achievable in approximately 90% of patients[4] Oral lesions typically respond to systemic treatment within weeks. Our patient showed clear improvement within 2–3 weeks, similar to cases treated with more aggressive regimens. At 1‐year follow‐up, she remains in remission with negative ANCA and no lesions, which is highly encouraging. Despite initial localization, long‐term follow‐up is essential due to GPA′s relapsing‐remitting nature [2, 14]. Relapses can occur at different sites, even in patients whose initial presentation did not involve the oral cavity. Such relapses are typically managed effectively with reinduction therapy, often utilizing rituximab [14]. Our patients favorable outcome suggests that a less intensive initial immunosuppressive strategy can be successful in selected localized cases, particularly in older patients with comorbidities, while carefully avoiding the potential toxicities of cyclophosphamide. The synergistic effects of systemic corticosteroids (deflazacort) and immunosuppressants (azathioprine) played a crucial role in achieving rapid and sustained resolution of the gingival lesions by targeting both the acute inflammatory response and the underlying autoimmune pathology [15].

**Figure 4:**
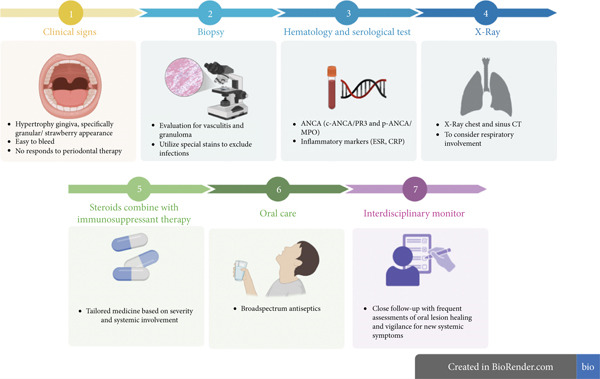
Clinical approach for GP.

However, as highlighted by Thompson et al., while oral lesions can be the presenting complaint, almost all patients eventually require systemic therapy, and even limited GPA has the potential to generalize, necessitating readiness to escalate therapy if needed [16]. Furthermore, the relapsing‐remitting nature of GPA necessitates long‐term follow‐up, as relapses can occur at different sites, even manifesting as new oral involvement, as demonstrated by Hanisch et al. where a recurrent gingival lesion responded effectively to rituximab. The key is to involve a rheumatologist to ensure prompt escalation of therapy if any systemic features emerge [14]. Based on this case and current evidence, we carried out a systematic clinical approach in line with the guidelines for granulomatous gingivitis, as shown in Figure 4.

## 4. Conclusion

This case report underscores the critical importance of including GPA in the differential diagnosis for atypical gingival hyperplasia or “strawberry gingivitis,” even in the absence of systemic signs. It reinforces the growing understanding that oral lesions, though rare, can serve as the sole initial manifestation of this potentially fatal autoimmune vasculitis.

Ultimately, increased awareness of oral GPA among oral health providers, who serve as crucial “gatekeepers” of the oral cavity, is essential to facilitate early diagnosis and appropriate treatment, thereby preventing progression to severe systemic disease. Implementing a standardized diagnostic and therapeutic protocol for oral GPA will further optimize patient outcomes and should be a focus for future documentation and research.

## Conflicts of Interest

The authors declare no conflicts of interest.

## Author Contributions

Sarwer Biplob was responsible for conceptualization, case management, and writing the original draft. Rahmi Amtha performed the writing of the complete manuscript, investigation, and data curation. Najla Nadiah contributed to the writing review and editing. Rachendra Pratama was responsible for editing the manuscript.

## Funding

No funding was received for this manuscript.

## Data Availability

The data that support the findings of this study are available on request from the corresponding author. The data are not publicly available due to privacy or ethical restrictions.
